# Mineralocorticoid receptor status in the human brain after dexamethasone treatment: a single case study

**DOI:** 10.1530/EC-21-0425

**Published:** 2022-02-11

**Authors:** Anne-Sophie C A M Koning, Philippe C Habets, Marit Bogaards, Jan Kroon, Hanneke M van Santen, Judith M de Bont, Onno C Meijer

**Affiliations:** 1 Division of Endocrinology, Department of Medicine, Leiden University Medical Center, Leiden, The Netherlands; 2 Einthoven Laboratory for Experimental Vascular Medicine, Leiden University Medical Center, Leiden, The Netherlands; 3 Department of Pediatric Endocrinology, Wilhelmina Children’s Hospital, University Medical Center Utrecht, Utrecht, The Netherlands; 4 Department of Pediatric Neuro-Oncology, Prinses Máxima Centrum, Utrecht, The Netherlands

**Keywords:** mineralocorticoid receptor, dexamethasone, neuropsychiatric adverse effects, cortisol, human pediatric brain

## Abstract

**Background:**

Synthetic glucocorticoids like dexamethasone can cause severe neuropsychiatric effects. They preferentially bind to the glucocorticoid receptor (GR) over the mineralocorticoid receptor (MR). High dosages result in strong GR activation but likely also result in lower MR activation based on GR-mediated negative feedback on cortisol levels. Therefore, reduced MR activity may contribute to dexamethasone-induced neuropsychiatric symptoms.

**Objective:**

In this single case study, we evaluate whether dexamethasone leads to reduced MR activation in the human brain. Brain tissue of an 8-year-old brain tumor patient was used, who suffered chronically from dexamethasone-induced neuropsychiatric symptoms and deceased only hours after a high dose of dexamethasone.

**Main outcome measures:**

The efficacy of dexamethasone to induce MR activity was determined in HEK293T cells using a reporter construct. Subcellular localization of GR and MR was assessed in paraffin-embedded hippocampal tissue from the patient and two controls. In hippocampal tissue from the patient and eight controls, mRNA of MR/GR target genes was measured.

**Results:**

*In vitro*, dexamethasone stimulated MR with low efficacy and low potency. Immunofluorescence showed the presence of both GR and MR in the hippocampal cell nuclei after dexamethasone exposure. The putative MR target gene *JDP2* was consistently expressed at relatively low levels in the dexamethasone-treated brain samples. Gene expression showed substantial variation in MR/GR target gene expression in two different hippocampus tissue blocks from the same patient.

**Conclusions:**

Dexamethasone may induce MR nuclear translocation in the human brain. Conclusions on *in vivo* effects on gene expression in the brain await the availability of more tissue of dexamethasone-treated patients.

## Introduction

Synthetic glucocorticoids are used extensively for their anti-inflammatory effects in many medical conditions, including immune diseases, different types of cancer, and in the treatment of edema in brain tumor patients (1, 2). A recent study from Denmark established a 3% annual prevalence of systemic glucocorticoid use that was stable over a period of 15 years (3). Although glucocorticoids are very efficacious, high-dose treatment can lead to severe neuropsychiatric effects like depression, anxiety, mania, delirium and even suicidality. Cognitive and memory impairments and sleep disturbances are also well-known side effects (4, 5). The high prevalence of the potentially severe adverse effects makes it important to study the biological mechanisms of action that underlie them, and this might help to decrease these adverse effects during glucocorticoid therapy.

Synthetic glucocorticoids target the glucocorticoid receptor (GR), one of the two receptor types for the naturally produced glucocorticoid hormone cortisol. Cortisol also acts via the mineralocorticoid receptor (MR) in many tissues including the brain (6), but synthetic glucocorticoids have a clear preference for GR over MR (7). Both receptor types are expressed in the brain. They are members of the nuclear receptor family and act as transcriptional regulators. Activation by cortisol leads to translocation of the receptors from the cytoplasm to the cell nucleus and subsequent interaction with DNA and proteins in the nucleus (8). However, MR and GR only partially overlap in their target sites at the chromatin (9) and they can mediate very different, sometimes opposite effects of cortisol on neuronal excitability, mood and cognition (6, 10). In relation to psychopathology, MR-mediated effects have been suggested to be protective based on genetic variants and pharmacological interventions (11, 12, 13).

Synthetic glucocorticoids via GR activation potently suppress the production of cortisol by the adrenal gland via activation of negative feedback mechanisms within the hypothalamic–pituitary–adrenal (HPA) axis (14, 15). High dosages of synthetic glucocorticoids will therefore lead to a situation of strong GR activation but, for lack of cortisol, lower activation of MR. While chronically elevated cortisol in Cushing’s disease may also lead to psychiatric complaints, next to the strongly activated GR, the reduced MR activity caused by synthetic glucocorticoids might contribute to the neuropsychiatric effects of dexamethasone (16). There is upcoming evidence that certain subgroups of childhood leukemia patients suffering from side effects from dexamethasone may benefit from cortisol add-on to refill and re-activate brain MR (17).

Here, we evaluated the validity of the ‘MR refill’ hypothesis in human pediatric brain tissue. We had the unique opportunity to investigate biological effects of dexamethasone in hippocampal brain tissue of an 8-year-old brain tumor patient, who experienced severe mental side effects in conjunction with his episodes of dexamethasone treatment and who died while on dexamethasone treatment. We assessed the expression and functionality of MR and GR, in part guided by the expression of recently identified MR-selective target genes in the rodent brain (18).

## Materials and methods

### Human brain tissue

Frozen and paraffin-embedded hippocampal tissue from an 8-year-old brain tumor patient was obtained from the biobank of the Amsterdam UMC. The patient was diagnosed with a primitive neuroectodermal tumor located in the pons. This pediatric patient received dexamethasone treatment to prevent tumor edema numerous times. Treatment was abrogated or dosages were lowered multiple times due to severe behavioral side effects but eventually was always reinstated. He was on continuous high-dose dexamethasone for nearly a year. The dexamethasone could only be tapered for a few weeks. In the end, the patient deceased while on a dexamethasone dose of 3.5 mg/day orally, 2 mg in the morning and 1.5 mg in the evening. The parents and treating physicians noted disinhibited behavior, sleep disturbances, increased appetite, mood swings and angry outbursts that seemed to be consistently occurring while the patient was on dexamethasone and decreased when dexamethasone was tapered. The mentioned complaints could not be explained by the location of the tumor, as these specific functions (behavior, sleep and appetite) are not located in the brainstem. He did not have tumor location in other parts of the brain, which excludes these complaints to be explained by the tumor and/or edema in other parts of the brain. Additionally, there were no signs of raised intracranial pressure, neither on MRI nor on ophthalmological examination. Also, a ventricular peritoneal drain was present to prevent hydrocephalus. The boy’s immediate cause of death was respiratory insufficiency due to extensive necrosis of the brain stem. The boy had received radiotherapy twice, and it is important to notice that the hippocampus and hypothalamus were not in the direct radiotherapy field. Only the brainstem was selectively irradiated with high dose.

Paraffin-embedded hippocampal tissue from two controls not taking glucocorticoids was obtained from the Department of Pathology of the LUMC. One control was a 5-year-old girl who died from a car crash and the second control was an 11-year-old girl who died from cardiac arrhythmia causing cardiac failure. Frozen hippocampal tissue from eight controls (five boys and three girls) who were not treated with glucocorticoids was obtained from the NIH NeuroBioBank at the University of Maryland, Baltimore, MD (Table 1). We have no information of which exact part of the respective hippocampus tissues we received. In two of the controls, the cause of death was asthma. According to the donor information, one control used salbutamol and over-the-counter (OTC) inhalers, and for the other control, medication use was not mentioned. The study was approved by METC VUmc and NIH NeuroBiobank. Consent has been obtained for the use of tissue according to institutional guidelines.

**Table 1:** Subject information of control tissues obtained from NIH NeuroBioBank.

Subject	Age (year)	Gender	PMI (hours)	Cause of death
#1	8	M	12	Drowning
#2	6	F	22	Smoke inhalation
#3	6	M	16	Drowning
#4	11	F	12	Asthma
#5	12	M	22	Cardiac arrhythmia
#6	12	M	13	Drowning
#7	12	M	15	Hanging
#8	13	F	17	Asthma

PMI, postmortem interval.

### Luciferase reporter assay

Human embryonic kidney cell line HEK293T were grown and maintained in Dulbecco’s modified Eagle’s medium (Gibco) supplemented with 10% fetal calf serum, 100 µg/mL penicillin and 50 µg/mL streptomycin. Cells were grown in a humidified incubator at 37°C and 5% CO_2_. HEK293T cells were seeded 80,000 cells in 500 µL serum-stripped medium in a 24-well plate. Cells were transfected using Fugene HD transfection reagent (Promega) with 25 ng TAT1-luciferase (19), 10 ng human MR expression vector, 1 ng CMV-renilla and 365 ng pcDNA. After 24 h, the medium was replaced and cells were stimulated with dexamethasone, aldosterone and cortisol in different concentrations ranging from 0.01 nM to 10 µM for 24 h. The firefly and renilla luciferase levels in the lysates were measured using dual luciferase assay (Promega), normalized by comparison with the control wells.

### Immunofluorescence staining

Paraffin-embedded hippocampal sections of 5 µm were mounted on coated slides (Starfrost), deparaffinized in histomount (National Diagnostics, Nottingham, UK) and rehydrated in a graded ethanol series. Antigen retrieval was performed in citrate buffer (10 mM, pH 6.0) in an autoclave at 120°C for 15 min. After cooling to room temperature, sections were washed with 0.1% PBS–Tween (PBST) and blocked with 5% BSA in PBS. Sections were incubated at 4°C overnight with rabbit monoclonal GR antibody (1:500, D6H2L, Cell Signalling technology) and mouse monoclonal MR antibody (1:500, MR1-18 1D5 and MR 4G5) (20) in 1% BSA/PBST. After washing, sections were incubated with AlexaFluor-488 labeled goat-anti-rabbit IgG and AlexaFluor-555 goat-anti-mouse IgG (1:250, Thermo Scientific) in 1% BSA/PBST for 90 min. Finally, sections were washed and nuclei were stained with DAPI (1:1000, Thermo Fisher Scientific) in PBS after which the sections were mounted with ProLong Gold anti-fade mounting medium (Thermo Fisher Scientific). Control sections were incubated with an equal amount of normal rabbit IgG (X0903, DAKO) as substitute for primary antibody. Additionally, sections were incubated without any primary antibodies to check for non-specific binding of the secondary antibodies. Confocal microscopy (Leica SP8) was used for the analysis of immunofluorescence (IF) staining using a 40× objective. ImageJ was used to calculate the percentage staining in the nuclei for each individual cell. Two pictures were taken from the tissue of the dexamethasone-treated patient and from two controls. In ImageJ, the region of interest was drawn, in which MR and GR are expressed and individual cell nuclei are clearly distinguishable. Within this region of interest, all cell nuclei were numbered based on the DAPI staining, and ImageJ software was used to calculate the percentage of red and green staining in all numbered nuclei. By comparing the distribution of percentage staining with visual evaluation, we established a cut-off value for the red and green staining, 10 and 7%, respectively. Staining above 10 and 7% for red and green signal, respectively, was considered a specific signal. The mean percentage of red/green-positive nuclei in two pictures of dexamethasone and controls tissues was calculated and used in the quantification.

### Quantitative-PCR

Frozen human hippocampal tissue was homogenized in TriPure (Roche) by using a hand-held rotary homogenization device for 30 s or until homogenization was achieved. Total RNA was isolated with chloroform, precipitated with isopropanol, washed with 75% ethanol and dissolved in nuclease-free H_2_O. The RNA concentration was determined with a NanoDrop spectrophotometer (Thermo Scientific) and 1 µg RNA was reverse-transcribed using random hexamers and M-MLV RT (Promega). RT-qPCR was performed in duplicates on 50× diluted cDNA (1 ng/µL) with final primer concentrations of 0.5 µM using iQ SYBR-Green supermix (BioRad) in a CFX96 real-time PCR machine (BioRad). Melting curve analysis was performed to ensure a single PCR product.

Two samples from the same hippocampal tissue block from the dexamethasone-treated patient were obtained from the neuropathology. Given the intrinsic vulnerability of comparing tissue from a single subject to reference tissues, these were processed independently, and from each sample, cDNA was made twice independently. We only had sufficient tissue from the controls to process RNA once. Expression of mRNA was evaluated for different glucocorticoid target genes. ‘Classical target genes’ that are often included in glucocorticoid research are known to be regulated by both GR and MR (18). These classical, mixed GR/MR target genes were FK506-binding protein 5 (*FKBP5*), glucocorticoid-induced leucine zipper (*GILZ*), period circadian clock 1 (*PER1*) and serum/glucocorticoid-regulated kinase 1 (*SGK1*). Furthermore, the receptor genes *NR3C1*(GR) and *NR3C2*(MR) were measured, and several target genes were measured that have been suggested to be specifically regulated by either MR or GR. These putative target genes were selected from previous ChIP-sequencing data in the rat hippocampus and from recently identified MR target genes in the mouse hippocampus (9, 18). The putative MR target genes were Jun dimerization protein 2 (*JDP2*), Suv3-like RNA helicase (*SUPV3L1*) and nitric oxide synthase 1 adaptor protein (*NOS1AP*). The putative GR-specific target gene was metallothionein 2A (*MT2A*). mRNA expression of genes of interest was normalized to mRNA expression of the housekeeping gene β-actin *in duplo*. We additionally validated the data using 18S primers on samples that were diluted to 500× in order to stay in the same Ct values range of the genes of interest (not shown). Primer sequences are listed in Table 2.

**Table 2:** Primer sequences used for quantitative real-time PCR on human hippocampal tissue.

Gene	Forward and reverse sequence	
*FKBP5*	Forward	TGGAAAGAAGTTTGATTCCAGTCAT
	Reverse	CATGGTAGCCACCCCAATGT
*GILZ*	Forward	CCAGCGTGGTGGCCATAGA
	Reverse	CACGCTCTAGCTGGGAGTTC
*PER1*	Forward	CACTCCTGCGACCAGGTA
	Reverse	TAGGGGGCCACTCATGTCT
*SGK1*	Forward	ACTCCTATGCATGCAAACACCC
	Reverse	AGAAGGACTTGGTGGAGGAGAA
*NR3C1*	Forward	TGCCTGGTGTGCTCTGATGA
	Reverse	CACATAGGTAATTGTGCTGTCCTT
*NR3C2*	Forward	GGGCAAAGGTACTTCCAGGATT
	Reverse	GTGCATCCCCTGGCATAGTT
*JDP2*	Forward	PROPRIETARY (Qiagen)
	Reverse	
*SUPV3L1*	Forward	TGTGCCATGTGACTTGGTGA
	Reverse	CCATCCTCTGGCTGGATCTCT
*NOS1AP*	Forward	ACATGCTCCAGCACATCTCC
	Reverse	GAGCCCATGGCGTTCTGT
*MTA2*	Forward	GCACCTCCTGCAAGAAAAGCTG
	Reverse	CGGTCACGGTCAGGGTTGTA

### Statistical analysis

For transfection studies, EC_50_ values were calculated using GraphPad Prism 7 software using a non-linear fit model. No formal statistical tests were performed on the results from the human brains, as this study only includes one patient. The q-PCR data were interpreted based on the expression levels in the dexamethasone patient relative to the average and s.d. of the control samples.

## Results

### Dexamethasone activates MR in HEK293T cells with low potency and low efficacy

**Figure 1:**
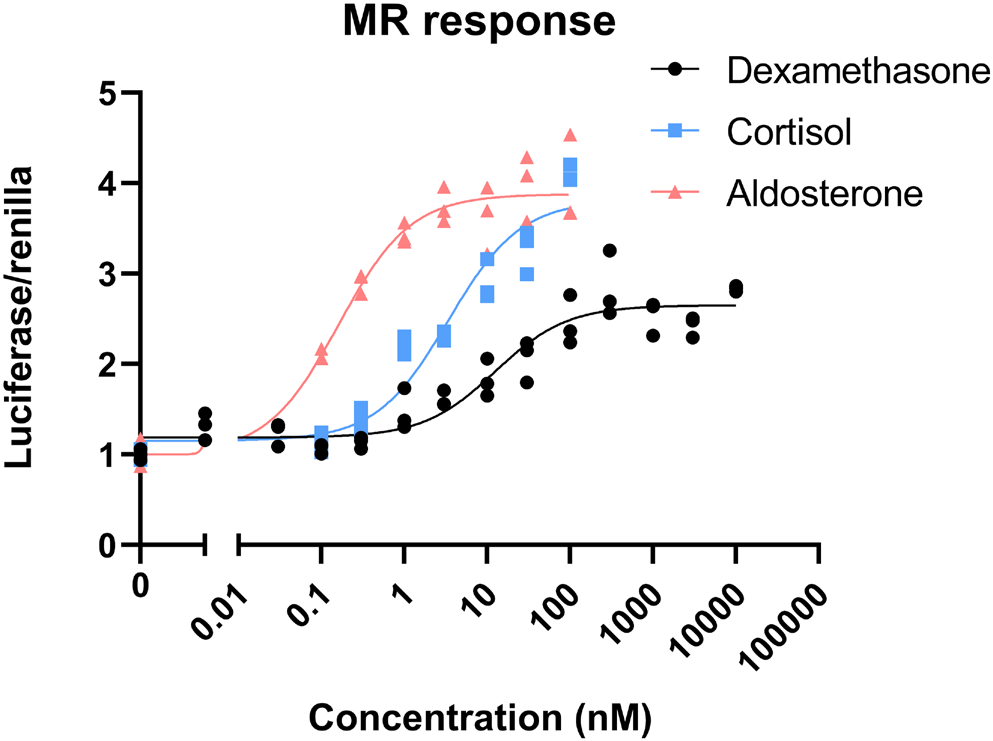
The effect of aldosterone, cortisol and dexamethasone on MR activity*in vitro*. Non-linear fit model for the concentration–effect in HEK293T cells transfected with a TAT1-luciferase reporter. Transactivation via hMR for aldosterone, cortisol and dexamethasone at different concentrations for 24 h.

Dexamethasone is known to bind MR but with very rapid kinetics, which is thought to render ligand–receptor complexes too unstable to be a strong MR agonist *in vivo* (21). To evaluate the ability of dexamethasone to activate MR under the continuous presence of ligand (steady-state concentrations), we generated a concentration–effect curve in MR-transfected HEK293T cells using an MR-sensitive luciferase reporter gene (Fig. 1). Increasing concentrations of the endogenous agonists aldosterone and cortisol enhanced the activation of MR with an EC_50_ of 0.16 and 3.4 nM, respectively. Dexamethasone was also able to activate MR, but higher concentrations were required. The EC_50_ of 12.5 nM demonstrates the lower potency of dexamethasone to activate MR. In addition, dexamethasone had lower efficacy, as it only reached about 70% of the maximal effect of aldosterone and cortisol. In HEK293T cells that were transfected with the only reporter and no MR or GR expression vector, no effect of hormones was observed (not shown). Overall, high concentrations of dexamethasone were able to activate MR in HEK293T cells, however, with low efficacy at the reporter gene even under the continuous presence of ligand.

### Cell nuclear presence of both GR and MR in dexamethasone-exposed human hippocampal tissue

**Figure 2:**
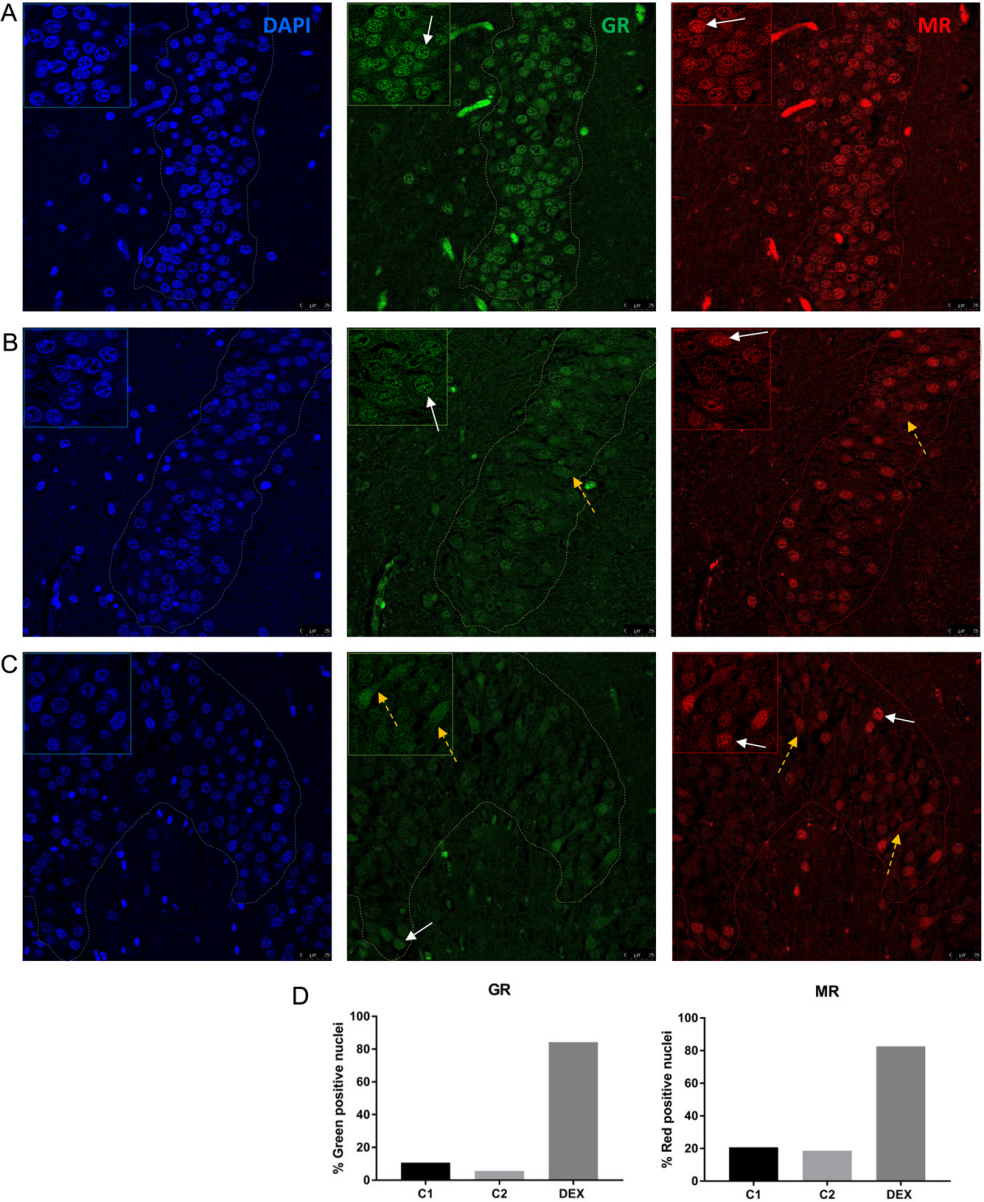
Cell nuclear localization of GR and MR in human hippocampal dentate gyrus (DG) region. Immunofluorescence staining of cell nuclei (blue), GR (green) and MR with the 1D5 antibody (red) in (A) tissue of the dexamethasone-treated patient and in (B) tissue from the 5-year-old control patient and (C) tissue from the 11-year-old control patient. (D) The percentage of GR- and MR-positive cell nuclei in the different tissues. White arrows show nuclear MR staining and yellow dotted arrows show cytosolic MR staining. In the left corner, a magnification is shown, and the dotted line represents the region of interest.

**Figure 3:**
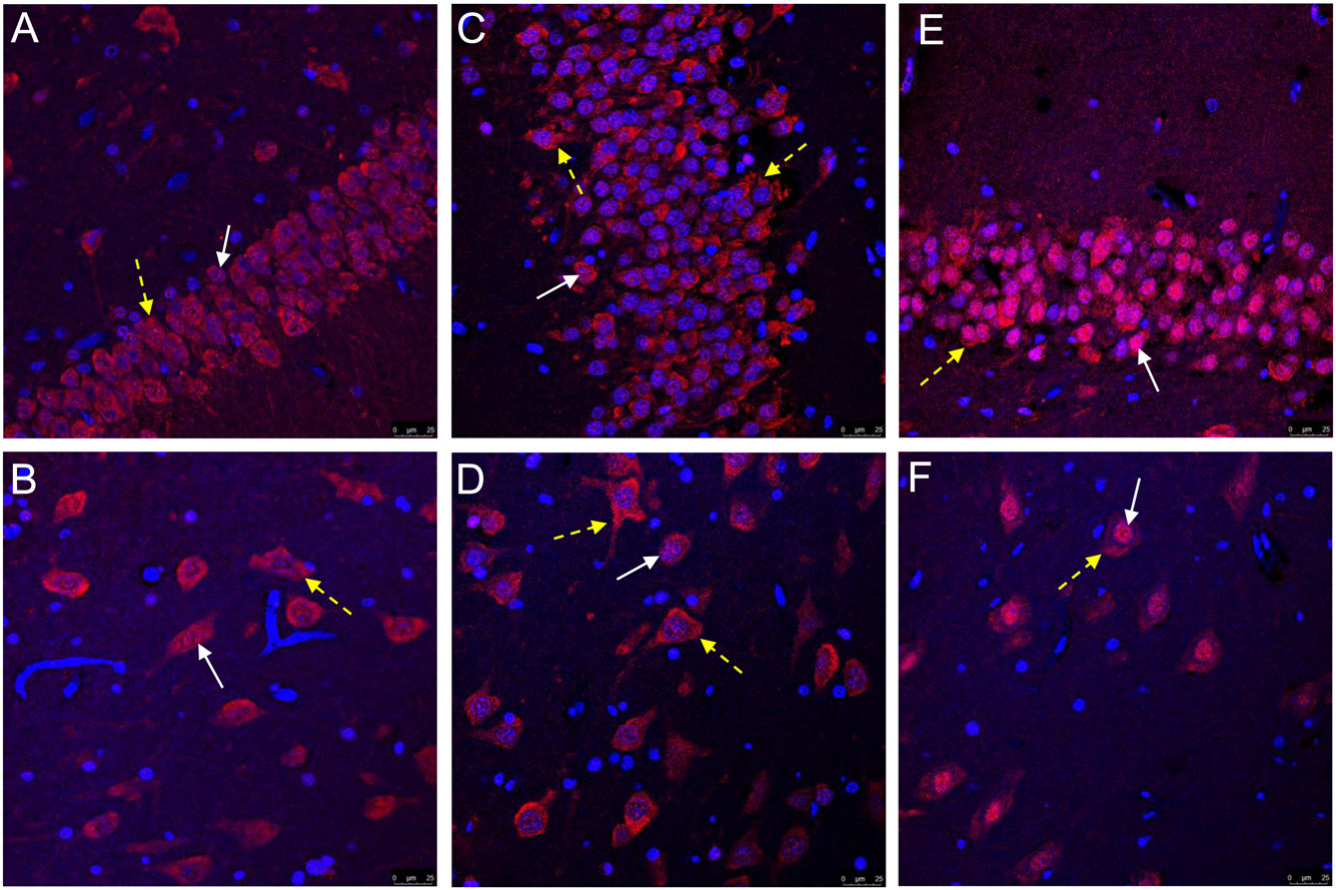
Cytosolic and nuclear MR staining in human hippocampal dentate gyrus (DG) and *Cornu Ammonis*4 (CA4) region. Immunofluorescence staining of cell nuclei (blue) and MR with the 4G5 antibody (red) in the DG (A, C, E) and CA4 (B, D, F) in tissue of the dexamethasone-treated patient (A, B) and tissue from the 5-year-old control patient (C, D) and tissue from the 11-year-old control patient (E, F). White arrows show nuclear MR staining and yellow dotted arrows show cytosolic MR staining.

We next visualized the subcellular distribution of the corticosteroid receptors in the human hippocampus. As expected, IF staining of GR in the dexamethasone-treated tissue showed clear nuclear localization (Fig. 2A), whereas in the two control tissues, the GR seemed more cytosolic (Fig. 2B and C). Nuclear staining was verified using DAPI as a nuclear marker. For MR, some nuclear staining was observed in the control tissues with the 1D5 antibody (Fig. 2B and C). Counter to our expectation, nuclear MR staining was also found in the dexamethasone-treated tissue (Fig. 2A). Quantification of nuclear GR and MR expression in both the patient and controls clearly demonstrated more GR- and MR-positive nuclei in the dexamethasone-treated tissue (Fig. 2D). This is not apparent for the MR 4G5 antibody, which showed a more cytosolic MR staining pattern (in line with earlier work (20)). In the dexamethasone-treated tissue and controls, this antibody showed both nuclear and cytosolic MR, suggesting that MR translocation was incomplete (Fig. 3). Overall, these data suggest that high-dose dexamethasone exposure causes nuclear translocation for both GR and MR, although for MR translocation, it is not complete. This is in line with the *in vitro* data where higher concentrations of dexamethasone were able to activate MR but at odds with binding studies in rodent brains (21).

### Gene expression levels in the human hippocampus: high variability between samples from the same subject

**Figure 4:**
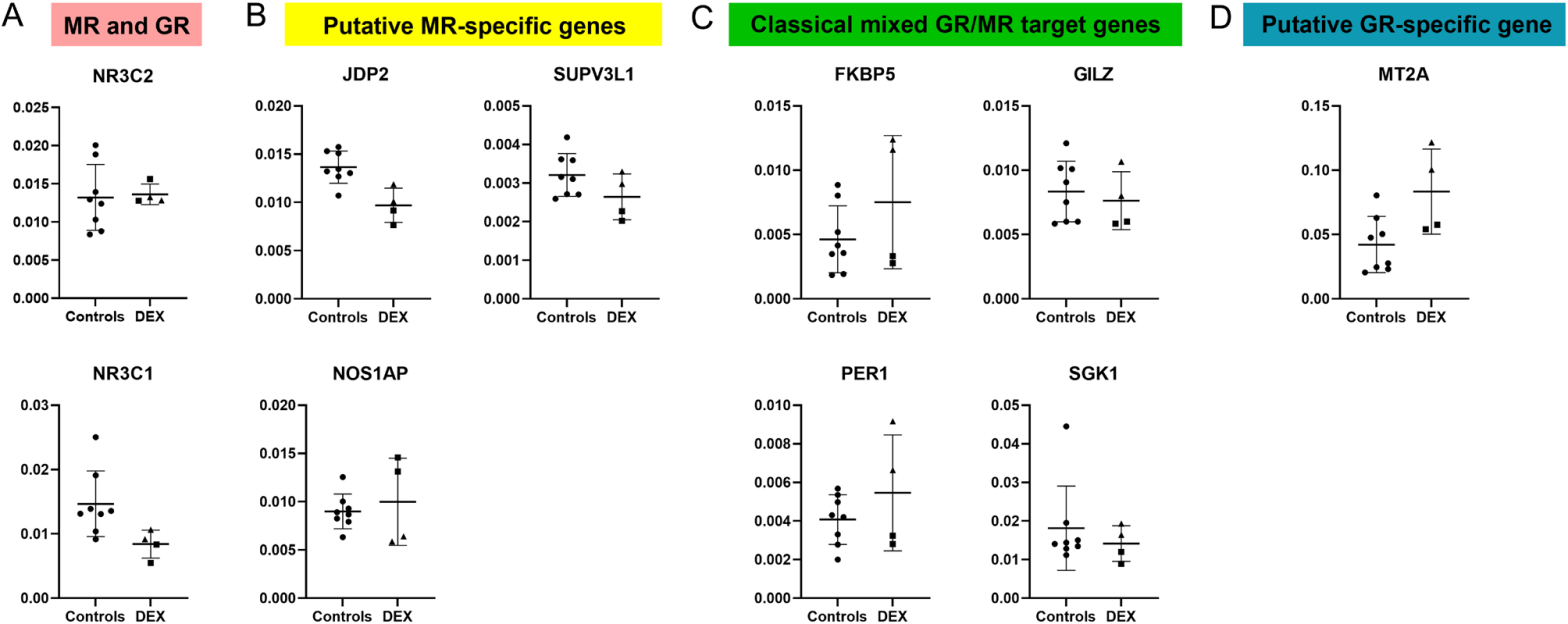
Expression of glucocorticoid target genes in human hippocampal tissue. Hippocampal mRNA levels of glucocorticoid target genes assessed in dexamethasone-treated tissue and controls with β-actin normalization. Gene expression of (A) *NR3C2* (MR) and *NR3C1* (GR), (B) putative MR-specific targets, (C) classical, mixed GR/MR targets and (D) putative GR-specific targets in *n* = 1 dexamethasone-treated patient vs *n* = 8 controls. Two tissue samples were independently processed with each having two independent cDNA samples (illustrated as squares and diamonds).

In order to find evidence for the transcriptional effects of dexamethasone on GR and MR in human hippocampal tissue, we investigated the functionality of the receptors in the dexamethasone-treated tissue and eight aged-matched control samples. To reduce the risk of chance findings in the tissue of the subject treated with dexamethasone, we determined mRNA expression in two separate blocks of hippocampal tissue, and for each block, we performed two independent rounds of cDNA synthesis. While cDNA from the same block of tissue resulted in reliable replication, there was a large variation between the different tissue blocks using β-actin normalization, in particular for some of the (putative) target genes. Expression levels of *NR3C2* were in the range of average expression observed in the controls, while *NR3C1* was expressed at the lower range (Fig. 4A). Expression levels of the best-established putative MR target gene, *JDP2,*were consistently low in the dexamethasone-exposed brain relative to the control samples. However, mRNA of *SUPV3L1* showed low expression in the first processed dexamethasone-treated tissue (as defined by ranking 9 or 8 in the whole sample set), but expression levels were clearly higher in the second tissue block (Fig. 4B). On average, the expression of the putative MR genes had a rank of 6.7 ± 3.2.

The expression of the classical, mixed GR and MR target genes *FKBP5*, *GILZ*, *PER1* and *SGK1* was also highly variable between tissue blocks. In the first tissue block, *GILZ* and *SGK1* mRNA were expressed at low levels, whereas in the second tissue, block expression of *FKBP5* and *PER1* was clearly in the higher range (Fig. 4C). Similarly, the expression of the putative selective GR target gene *MT2A* was high for second dexamethasone-treated tissue sample (Fig. 4D). These high values are more in line with what may be expected from GR target genes after high dexamethasone, but the potential role of MR precludes a straightforward predication (18). Average ranking of these genes that are GR sensitive was 3.2 ± 1.9.

Altogether, these data demonstrate a rather disconcerting variation in hippocampal expression of target genes in the two tissue blocks from the same patient (see discussion). Although in the control tissue similar technical variation may have been present, the higher number of samples from different subjects should lead to a more reliable estimate in which technical and biological variation are both present. Of note, 18S normalization tended to show less extreme variation between the two tissue samples with respect to the extreme ‘batch effect’ that was observed for, for example, *FKBP5*. However, relative average expression levels between case and controls were essentially identical using 18S normalization (not shown).

## Discussion

The aim of the current single case study was to evaluate the hypothesis that dexamethasone treatment leads to reduced MR activation in the human brain, in the context of the ‘MR refill’ concept to reduce psychiatric side effects of synthetic glucocorticoids treatment by add-on of cortisol. This was possible because of the availability of a unique hippocampal tissue from an 8-year-old brain tumor patient, who died during treatment with dexamethasone, only hours after the last administration. The boy had suffered from neuropsychiatric effects that accompanied the many dexamethasone treatments he had received. In this study, we confirm the ability of dexamethasone to activate MR *in vitro*, although with low potency and low efficacy. In human hippocampal tissue treated with dexamethasone, we demonstrate nuclear translocation of GR and MR. Even if translocation was incomplete for MR, the data suggest that dexamethasone, hours after a very high dose, is able to bind to MR sufficiently to induce partial translocation. Although the pharmacokinetics *in vivo*is uncertain, the data are consistent with the known *in vitro* effects of dexamethasone that we also observed.

Unfortunately, we observed highly variable mRNA expression levels of many MR and or GR target genes in dexamethasone-treated tissue, as compared to control tissue. The low expression of the characterized mouse MR target gene *JDP2* is supportive of our hypothesis but should be interpreted with caution given the highly variable expression of established GR targets. This argues that the comparison of this single case does not permit a conclusion. Even with tight data, strong conclusions based on a single case would be challenging. The different results in the two samples from the same subject might be explained by the differential presence of different hippocampal cell types and subregions. A recent single-cell transcriptome data analysis of mouse hippocampus showed that the expression of GR target genes is cell-type specific (under basal conditions) (22). For example, *Fkbp5* seems to have highest expression in the glutamatergic neurons. However, if we would know the exact origin of the tissue – region and its cell types – we could get much better detail of the expression of the genes of interest. Normalization with 18S tended to show less-extreme variation between the tissue samples; however, it yielded an identical value average to be representative in the hippocampus as a whole.

Our assumption of MR underactivation after dexamethasone hinges on the depletion of endogenous cortisol by dexamethasone. To our knowledge, there are no reports of desensitization of the negative feedback on the HPA axis as a consequence of chronic glucocorticoid exposure. Even if GR is known to suppress its own expression via a negative glucocorticoid response element (GRE) (23), Cushing’s patients and for example, AdKO ‘Cushings’ mice’ show sustained GR-mediated effects even after very long-term (in this case: endogenous) glucocorticoid overexposure (24). The dexamethasone-exposed tissue represents a context of high GR occupation. The lower expression range of GR mRNA would be in line with high GR activity in the dexamethasone-exposed tissue.

Dexamethasone is used clinically in particular in oncology, neurosurgery and immunology and has been studied in relation to brain corticosteroid receptors for almost 50 years (25). While its potent suppressive effect on the HPA axis primarily takes place at the level of the pituitary, dexamethasone at high doses penetrates the blood–brain barrier (26). When administered in low doses (in absence of a functional blood–brain barrier), it clearly and selectively binds brain GRs (27), and hence, nuclear GR staining was expected in the hippocampus of the dexamethasone-treated subject. However, the nuclear presence of MR also suggests some level of activation. From kinetic experiments, it is known that dexamethasone has a comparable association binding rate for MR as corticosterone but has a very high dissociation rate (21). As a result, an unstable binding complex is formed, which is probably why MR is not activated *in vivo*under non-steady-state conditions. Our data under *in vitro* steady state conditions likely reflect these binding kinetics. Based on our current (translocation) data, we hypothesize that under conditions of intermittent dexamethasone treatment, there may in fact be a partial temporary activation of MR. Another possibility is nuclear translocation of a heterodimer complex, which might consist of a GR activated by dexamethasone and a weakly/transiently bound MR (28). This complex could translocate to the nucleus, but whether the binding, in both cases, is strong enough to actually regulate gene transcription remains to be investigated (when brain tissue from more dexamethasone/glucocorticoid-treated subjects is available).

As discussed in detail elsewhere (16), psychological adverse effects of dexamethasone could be caused by GR overactivation, MR under activation or by a disbalance in MR:GR activity. It was already tentatively concluded that re-activating MR by cortisol add-on treatment may alleviate the psychological adverse effects of dexamethasone in the subgroup of patients with childhood leukemia (17). Re-activation of MR – in the context of high GR activation – seems to be an important goal for the improvement of glucocorticoid therapy by reducing psychiatric side effects.

The alternative explanation for a beneficial effect of cortisol would be that cortisol-activated GR exerts different effects from dexamethasone-activated GR. Given the strong suppression of the HPA axis after dexamethasone, and the differences between cortisol-MR and dexamethasone-MR, we favor the option that MR mediates favorable effects of cortisol, but – again – the current data on human hippocampal gene expression does not allow us to differentiate between these options. It will be necessary to replicate our findings in samples from other individuals, to substantiate the lower MR activity in the human brain after synthetic glucocorticoid treatment, in relation to psychiatric side effects that are regularly observed with this treatment.

## Declaration of interest

The authors declare that there is no conflict of interest that could be perceived as prejudicing the impartiality of the research reported.

## Funding

This work is funded by The Netherlands Organisation for Health Research http://dx.doi.org/10.13039/100005622 and Development (ZonMw http://dx.doi.org/10.13039/501100001826): grant project number 95105005.
